# Mobile-based ecological momentary assessment and intervention: bibliometric analysis

**DOI:** 10.3389/fpsyt.2024.1300739

**Published:** 2024-02-26

**Authors:** Hongfan Yin, Hanjing Zhu, Jia Gu, Hengwei Qin, Wenjing Ding, Ningyuan Guo, Jingjing Fu, Yan Yang

**Affiliations:** ^1^ School of Nursing, Shanghai Jiao Tong University, Shanghai, China; ^2^ Department of Urology, Renji Hospital, Shanghai Jiao Tong University, Shanghai, China; ^3^ Department of Sports Medicine, Huashan Hospital, Fudan University, Shanghai, China; ^4^ Reference Department, Library of Shanghai Jiao Tong University School of Medicine, Shanghai, China; ^5^ Department of Nursing, Renji Hospital, Shanghai Jiao Tong University, Shanghai, China

**Keywords:** mobile technology, ecological momentary assessment, ecological momentary intervention, ambulatory assessment, bibliometric analysis

## Abstract

**Objective:**

The aim of this article was to review existing documents in the field of mobile-based EMA and EMI, provide an overview of current hot topics, and predict future development trends.

**Methods:**

We conducted a bibliometric study on mobile-based EMA and EMI publications that were collected from the Web of Science Core Collection database. Biblioshiny and CiteSpace were utilized to analyze scientific productions, leading sources, authors, affiliations, documents, research hot topics, keywords, and trend topics.

**Results:**

A total of 2222 documents related to EMA and EMI published between 1992 and 2023 were retrieved. In recent years, scholarly publications have generally increased in mobile-based EMA and EMI research, particularly in the last decade. JMIR mHealth and uHealth (n=86), as well as JMIR (n=73), showed the highest number of publications. The United States (n=1038), Germany (n=218) and Netherlands (n=175) were leading countries. Regarding keyword co-occurrence and trend topics analysis, mental health, health behaviors, and feasibility were hot topics in mobile-based EMA and EMI research. Future research trends included using EMA for tailoring EMI, just-in-time adaptive interventions (JITAI), and digital phenotyping.

**Conclusion:**

This bibliometric study on mobile-based EMA and EMI is a valuable resource for understanding the field’s evolution and future trends. Our analysis indicates that EMA and EMI have great potential in health behaviors and mental health, but implementation should consider feasibility and reactivity issues carefully. Emerging trends include EMA-tailored EMI, JITAI, and digital phenotyping. In the future, strengthening multidisciplinary cooperation will be necessary to promote the continued development of the field.

## 1 Introduction

Ecological momentary assessment (EMA) involves various research techniques that capture data repeatedly, in real-time, and in real-life experience (1).EMA can measure natural phenomena that occur and change over time, with the strength of improving ecological validity and reducing recall bias (1). Using EMA has led to new insights into the dynamic associations among behaviors, contexts, and psychological states and may help inform the achievement of real-time and tailored interventions and treatments (2).

Ecological momentary intervention (EMI) is an extension of EMA, which facilitates the provision of real-time interventions in real-world settings (3, 4). Participants may receive behavioral or psychological support daily, practicing new behaviors and skills anytime and anywhere (5–7). EMI can also validate the efficacy of clinical interventions or treatment programs using real-world data (4). By leveraging the benefits of EMI, interventions and treatments can be systematically applied in real-world settings, increasing the accessibility and impact, making them more widely accessible, and increasing their overall impact (4).

Mobile technologies are a robust methodology that can support the implementation of EMA and EMI (1). Integrating mobile technology into EMA and EMI can provide researchers and clinicians with a more convenient and flexible way of conducting assessments and interventions. Commonly used mobile technologies, including smartphones and handheld devices, offer the ability to assess and intervene in any location at any time, enabling more comprehensive data collection and treatment implementation (4). Furthermore, mobile technologies can improve the efficiency of assessment, treatment, and care (8). For example, EMA, in combination with ambulatory physiological monitoring devices, can capture ambulatory physiological data, including heart rate and skin conductance, as well as context-sensitive data, which can offer a more accurate and complete view of the participant’s experience (9, 10). Additionally, mobile technologies enable EMI to be tailored to the participant’s characteristics and feedback and can be provided at the specific time when the participant needs the most support, increasing their satisfaction and willingness to engage with the intervention (5). By simplifying repetitive interventions and treatments, mobile technologies can also improve the efficiency of care, allowing medical staff to spend their time more effectively. This reduces duplication of work and improves the quality of treatment and care provided to patients (4).

Over the past few decades, research on mobile-based EMA and EMI has flourished in various fields, including mental health (11), dietary behaviors (12), physical activity (13), substance use (14), and addictive behaviors (15, 16); and across diverse age groups, ranging from children to the elderly (17–21), resulting in a plethora of publications each year (22–24). While there have been significant developments in mobile-based EMA and EMI research, there still needs to be more comprehensive perspectives to synthesize and summarize the current state of the field effectively. The bibliometric analysis provides a quantitative and structured method for reviewing and describing research trends and knowledge structures. Therefore, we conducted a bibliometric study of this field to inform researchers about the latest developments and trends in this rapidly evolving field.

## 2 Methods

### 2.1 Data collection

A comprehensive search was conducted on the Web of Science Core Collection database (WOSCC) to retrieve documents in the Science Citation Index Expanded (SCI-EXPANDED). The following terms and search strategies were used: (TS=“mobile” OR TS=“mobile device*” OR TS=“mobile technology” OR TS=“mhealth” OR TS=“ehealth” OR TS=“telehealth” OR TS=“mobile health” OR TS=“smartphone” OR TS=“app” OR TS=“application*” OR TS=“telemedicine” OR TS=“smartphone” OR TS=“phone”) AND (TS=“ecological momentary” OR TS=“ecological momentary assessment” OR TS=“ecological momentary intervention” OR TS=“ambulatory assessment” OR TS=“experience sampling”). The time period of the documents was chosen from the creation of the database to June 30, 2023. We retrieved a total of 2509 articles. After excluding 11 non-English language articles, 129 meeting papers, 78 unspecified, 45 abstracts, 11 books, 7 letters, 3 corrections, and 3 data papers, we finally obtained 2222 articles. The detailed information of all selected documents was downloaded in BibTeX format.

### 2.2 Bibliometric analysis

A bibliometric analysis of the literature on mobile-based EMI and EMA was conducted using the Bibliometrix package (version 4.0) in R (25). Initially, we extracted five BibTeX files from WOS, each containing “Full Record and Cited References” data. It’s worth noting that WOS limits each file to a maximum of 500 records, and since we had a total of 2222 records, we needed to split them into five separate files. Subsequently, we merged all of these files into a single text file using Apple Terminal Services (2.12.7). Following that, we utilized R Studio software to run the R package “Bibliometrix”. Within the interactive interface, we imported the prepared text file into the “import” section of Biblioshiny to perform Bibliometric analysis. This study examined several key bibliometric indicators, including annual document production, leading journals and authors, leading affiliations, leading countries or regions, international cooperation analysis, leading documents, keywords analysis, and trend topics analysis, to reveal the development and intellectual structure of the field. Relevant bibliometric indicators, such as density, distribution, and centrality, can be referenced for detailed definitions and calculation methods as provided in the Bibliometrix documentation, available at https://www.bibliometrix.org/vignettes/Introduction_to_bibliometrix.html. This source is an invaluable tool for understanding and applying these metrics in bibliometric analysis, offering comprehensive guidance on leveraging Bibliometrix for scholarly research evaluation and trend analysis. Biblioshiny (25), an interactive web-based application that provides a user-friendly interface to Bibliometrix, was used.

CiteSpace (version 5.7. R3; College of Information Science and Technology, Leisel University, USA), a software tool developed to visualize and analyze trends and structures in scholarly publications, was used to conduct a burst detection analysis to discover the most vigorous and impactful research areas in mobile-based EMI and EMA (26).

## 3 Results

### 3.1 Publication output and growth rate

**Figure 1:**
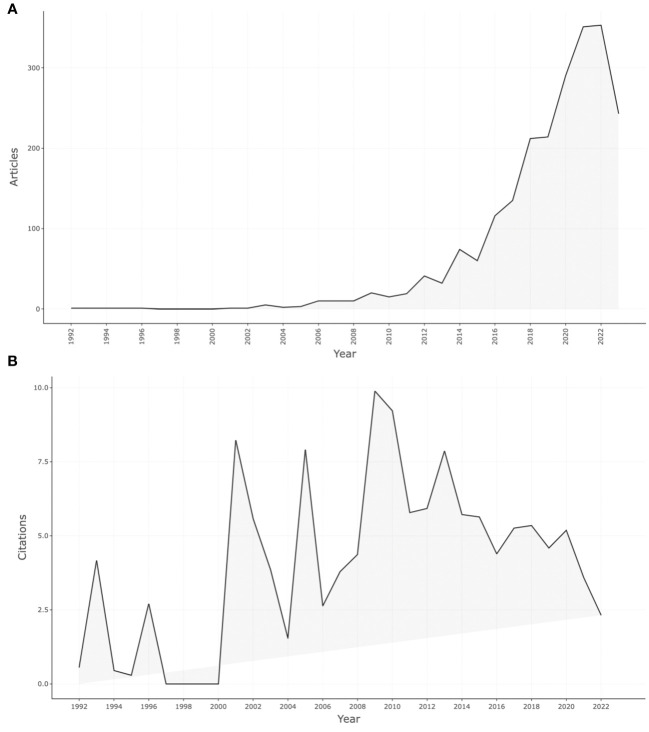
Annual publications of mobile-based EMA and EMI from 1992 to 2023 **(A)**; Average citations per year of documents in mobile-based EMA and EMI **(B)**.

The included documents were published from 1992 to 2023. A total of 2,222 documents were analyzed, written by 8,245 scholars, resulting in an average of 3.71 authors per document. These documents were published across 750 sources (**Table 1**). **Figure 1A** shows a broad growth in scientific publications in recent years, particularly within the last ten years. Notably, 2021 and 2022 generated the highest amount of publication output, with 351 and 353 documents published. Furthermore, mean citations per year per document were also calculated, and documents published in 2009 had the most notable mean total citations per year (**Figure 1B**).

**Table 1:** Detailed information of the included documents.

	Description	Results
**Main information**	Time-span	1992-2023
	Documents	2222
	Sources (Journals, Books, etc)	750
	References	77421
Document types		
	Article	1956
	Review	164
	Others (early access, clinical trials, editorial materials, case reports)	102
Authors collaboration		
	Authors	8245
	Single-authored documents	49
	Co-Authors per documents	5.7
Collaboration index		
	Annual Growth Rate %	19.93
	Average citations per doc	20.77

### 3.2 Leading journals

**Figure 2:**
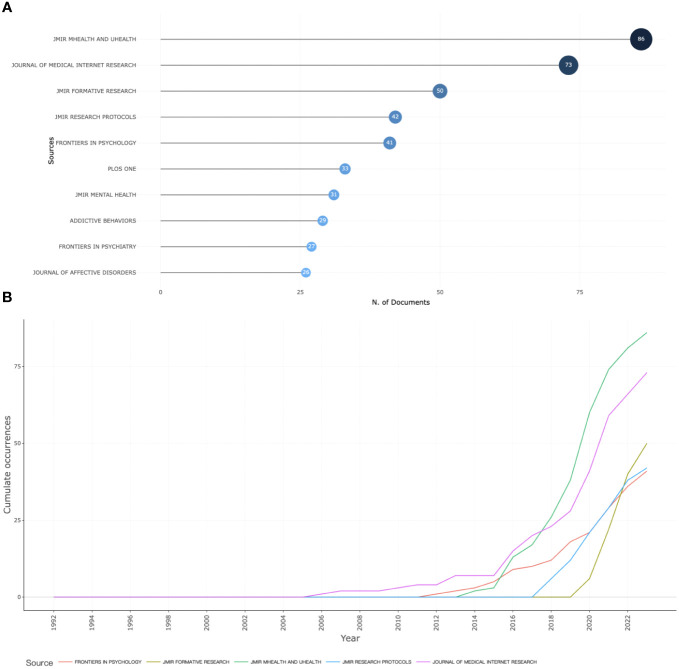
Most relevant sources in mobile technology-based EMA and EMI **(A)**; Dynamics of the top five sources **(B)**.

The journal that has published the most documents in the field was JMIR mHealth and uHealth (n=86), followed by Journal of Medical Internet Research (JMIR) (n=73), and JMIR formative research (n=50), as shown in **Figure 2A**. The dynamics of the top five sources are displayed in **Figure 2B**, which revealed that, in the beginning, there were very few sources in this field. However, all sources demonstrated a yearly growth trend. Notably, JMIR mHealth and uHealth started relatively late but exhibited rapid growth, with 86 total documents published between its first publication in 2014 and 2023. Journal of Medical Internet Research also experienced substantial growth, from the first document published in 2006 to 73 documents published in 2023.

### 3.3 Leading authors

**Figure 3:**
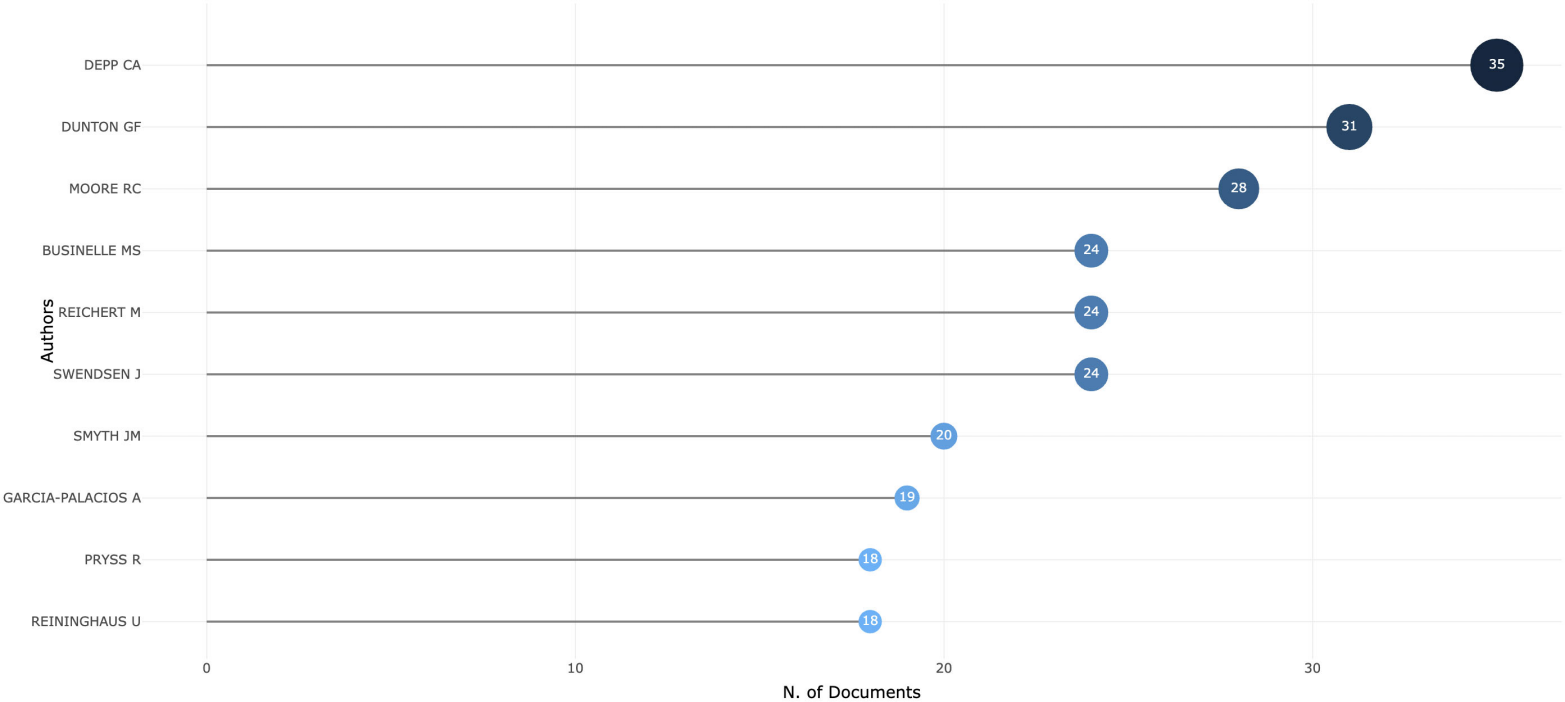
Top 10 most relevant authors.

As shown in **Figure 3**, the leading authors publishing the greatest number of documents have been identified. Colin A Depp from the University of California, San Diego, authored the most publications with 35. Genevieve DuntonF, affiliated with the University of Southern California, was the second most prolific author, having published 31 documents. Raeanne C. Moore, associated with University of California, San Diego, was ranked third with 28 publications.

### 3.4 Leading affiliations

**Figure 4:**
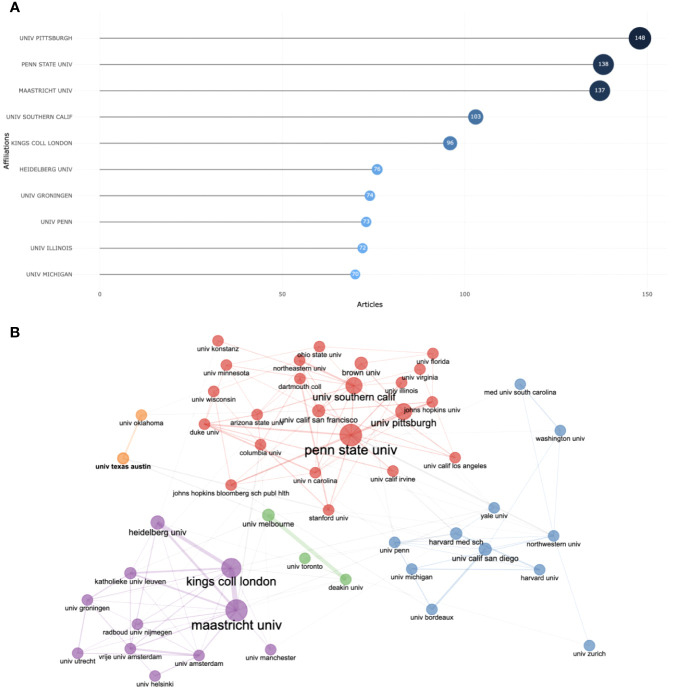
Top 10 most relevant affiliations **(A)**; Institutions collaboration network. **(B)**.

As shown in **Figure 4A**, the University of Pittsburgh was the top-ranking affiliation for research in this area, with a total of 148 publications. Penn State University followed with 138 publications, and the Maastricht University ranked third with 137 publications. To enhance the understanding of inter-institutional relationships, the methodologies of coupling and clustering are employed in the analysis. In the context of institution-specific investigations, “coupling” delineates the connection established by two or more research institutions through the mutual citation of identical scholarly literature. Conversely, “clustering” involves the aggregation of these research institutions based on the intensity of their coupling, thereby forming distinct research clusters or networks (27). This organization facilitates a comprehensive comprehension of the structure and dynamics underlying research collaborations. Within this analytical framework, each node symbolizes a distinct institution, with the node’s color denoting the cluster to which the institution is affiliated. **Figure 4B** shows the leading institutions divided into 7 clusters, each represented by a different color. This indicates the presence of connections among the institutions. Furthermore, nodes and links sharing identical colors suggest that a significant proportion of the papers cited by these universities originate from institutions within the same or closely associated clusters. This pattern also intimates that authors tend to collaborate and communicate predominantly within their proximate geographical regions and communities. Kings College London and Maastricht University collaborated vigorously, as did The University of Melbourne and Deakin University.

The dynamics of the top 5 affiliations are displayed in **Supplementary Figure 1**. Penn State University and the University of Pittsburgh are among the institutions that started early and have seen a swift increase in the number of published articles. Penn State University’s output grew from 3 articles in 2002 to 138 articles by 2023, while the University of Pittsburgh went from publishing 3 articles in 2003 to 148 articles by 2023. Although Maastricht University commenced its publishing activities later than the first two, with its first article published in 2006, its growth has been rapid, reaching a total of 137 publications by 2023.

### 3.5 Leading countries

**Figure 5:**
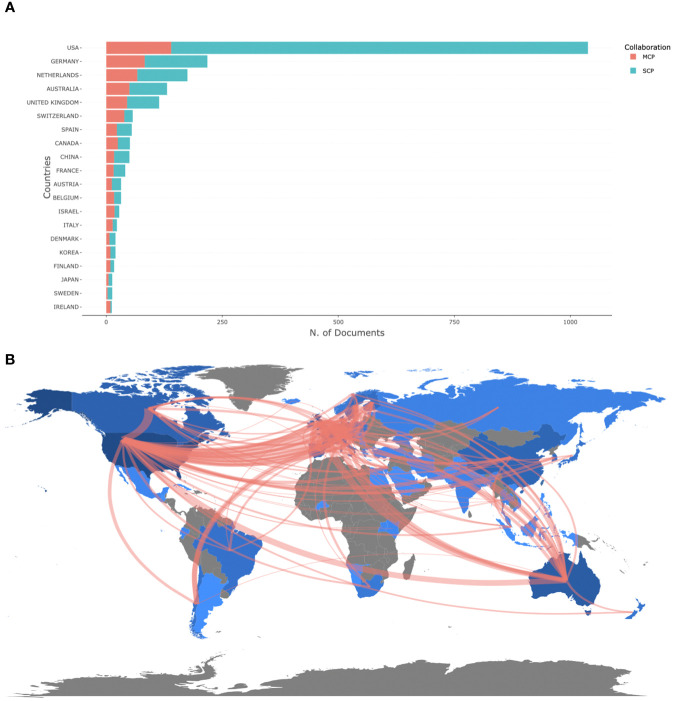
The leading countries according to the amount of documents the author’s country tallied. SCP: Single Country Publications; MCP: Multiple Country Publications **(A)**; The international cooperation network map **(B)**.

Forty-five countries in total were incorporated in the completed documents. Regarding the quantity of publications, the United States ranked first (n=1038), with Germany (n=218) and Netherlands (n=175) coming in second and third. The United States produced the greatest single country publications (n=898), followed by Germany (n=135) and Netherlands (n=108). Multiple country publications were led by the US (n=140), followed by Germany (n=83) and the Netherlands (n=67) (**Figure 5A**; **Supplementary Table 1**). In terms of country-to-country cooperation, as shown in **Figure 5B** and **Supplementary Table 2**, the collaboration between the Netherlands and the United Kingdom was the strongest (n=51), followed by the United States and the United Kingdom (n=50), and Germany and Netherlands (n=46).

### 3.6 Leading documents

**Figure 6:**
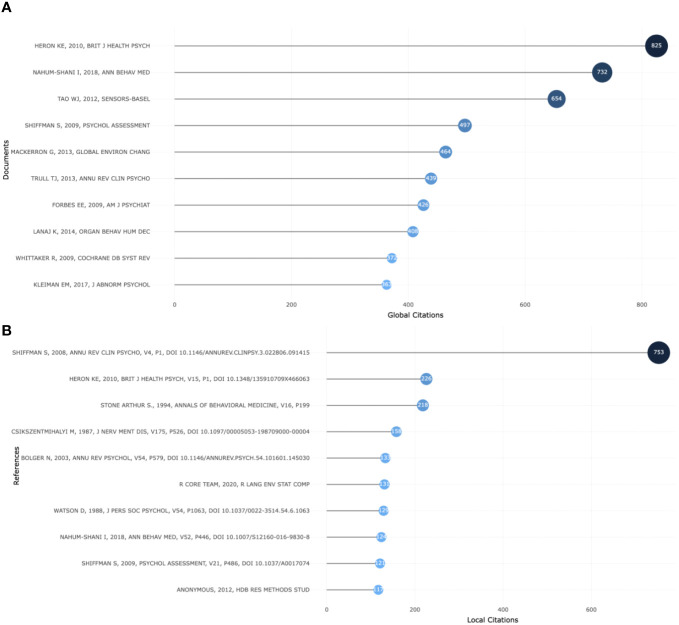
The top most-cited documents globally **(A)**; The top most cited references locally **(B)**.

Global citations can represent the impact of documents in the comprehensive database. **Figure 6A** displays the top 10 most cited documents globally. Notably, Heron’s 2010 article (4) was the most cited global article, with 825 citations, while Nahum-Shani’s 2018 (28) article was the second most cited global document, with 732 citations. Another noteworthy publication is the 2008 article by Shiffman (1), which had the highest number of citations as a reference at 753, according to **Figure 6B**. These highly cited documents serve as invaluable references for researchers to build upon to advance the field further.

### 3.7 Keyword analysis

**Figure 7:**
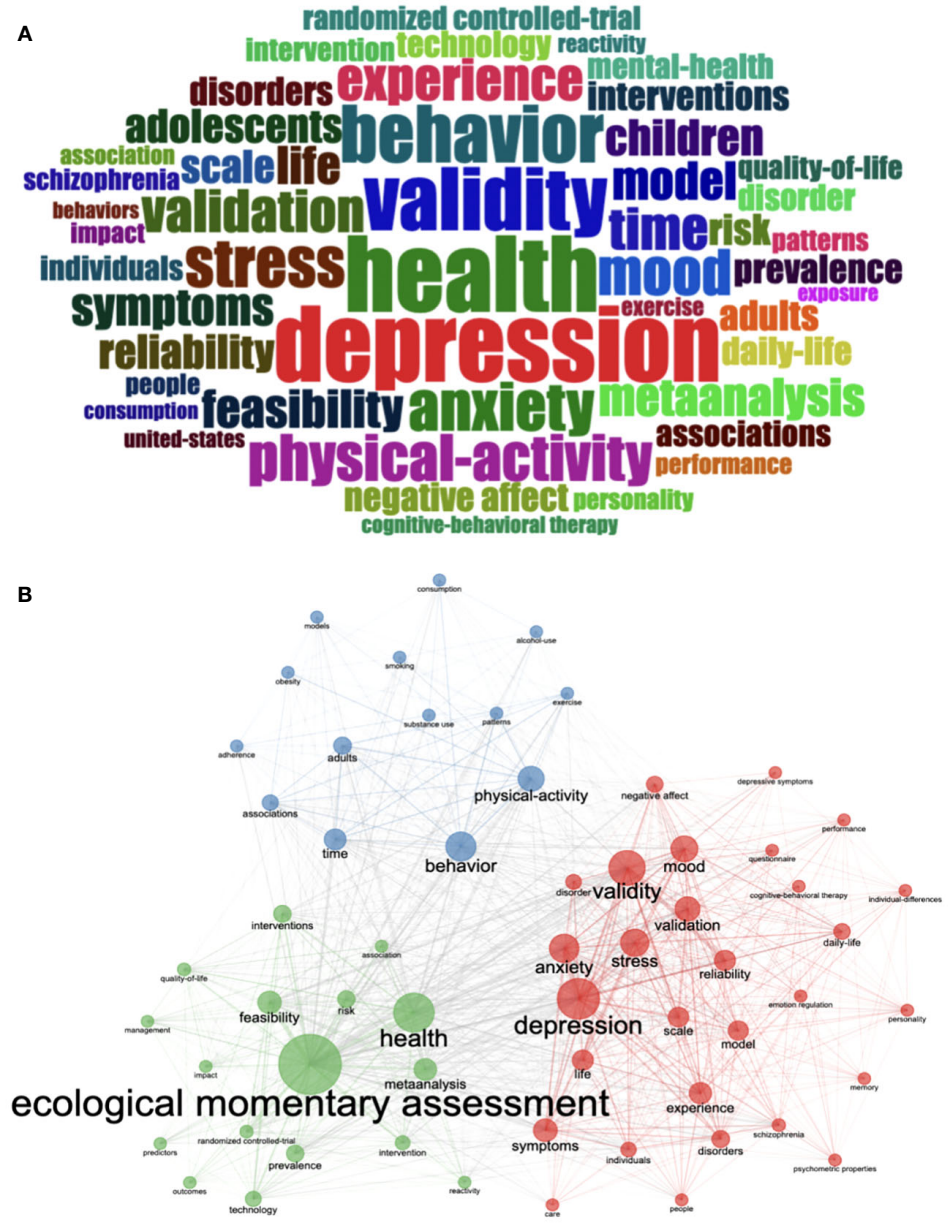
Word Cloud of Keywords Plus **(A)**. Keyword co-occurrence map **(B)**.

Three thousand nine hundred and forty-six keywords plus from all the selected publications were analyzed in the keyword analysis. The top 10 most widely used keywords in this field were ecological momentary assessment (403), health (219), depression (218), validity (181), behavior (165), stress (141), anxiety (140), physical activity (134), mood (132) and validation (123) (**Figure 7A**; **Supplementary Table 3**). To further explore the association between keywords, cluster analysis was further used in the analysis. Cluster analysis of keywords involves grouping keywords by quantifying their co-occurrence frequencies, with keywords in each colored cluster indicating that they are mostly used together (27). The larger the node, the more frequently the keyword is used. The most commonly used keywords were plotted as 3 clusters (**Figure 7B**). Cluster 1 (red) included depression, validity, stress, anxiety, mood, disorders, schizophrenia, cognitive-behavioral therapy, and depressive symptoms. Cluster 2 (blue) included behavior, physical-activity, time, exercise, consumption, smoking, obesity, and alcohol-use. Cluster 3 (green) included feasibility, interventions, prevalence, reactivity and technology.

### 3.8 Trend topics

**Figure 8:**
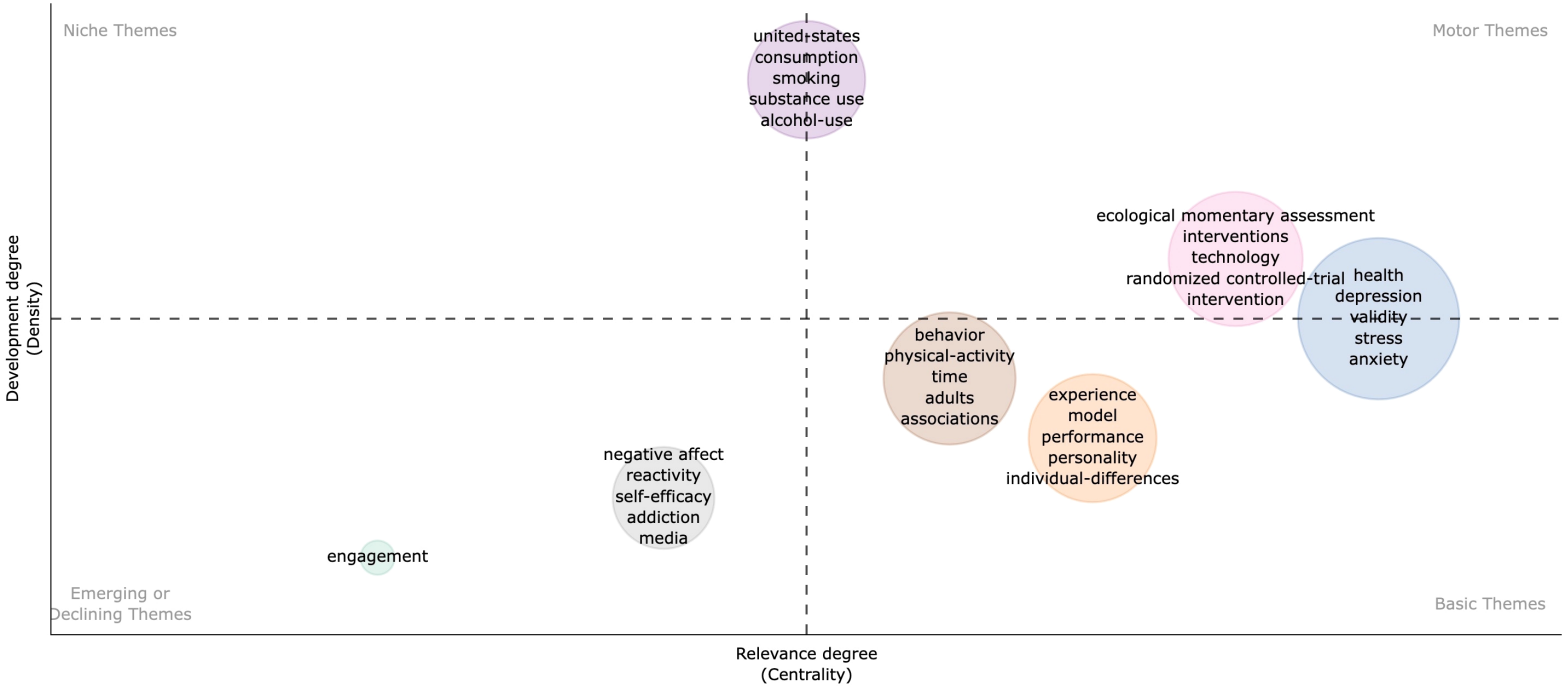
Thematic map of Keywords plus.

Based on the keyword co-occurrence network, the centrality and density of each cluster are calculated and the theme map is drawn, with each circle representing a cluster of the same color. Centrality indicates the proximity of a topic to other topics, while density indicates the maturity of a topic. In the theme map (**Figure 8**), the first quadrant (upper right) represents motor themes, which are both significant and fully developed. The second quadrant (upper left) represents niche themes, which are fully developed but have yet to be relevant to the field. The third quadrant (lower left) includes emerging or declining themes, representing marginal ideas that need to be well-developed, may have recently emerged, or are on the verge of disappearing. The fourth quadrant (lower right) includes basic themes that are essential to the field but have yet to be fully explored.

Health-related behaviors such as smoking, substance abuse, alcohol consumption, as well as psychological aspects like depression, anxiety, and stress, along with technology-related assessments and interventions, were important and well-developed topics for mobile-based EMA and EMI. Specifically, the United States also appears in this topic, reflecting the leadership position of the United States with numerous papers published in the field.

Exploring individual differences and personality traits were crucial topics in the current field and needed further development, as they were located in quadrant 4 of the theme map. Although these topics were essential, they were not yet well-developed, and increased research was warranted to explore their potential in various health domains.

In the context of EMA and EMI applications, issues such as engagement and reactivity fell into the emerging or declining categories. These categories represented marginal ideas that required further development and exploration.

**Figure 9:**
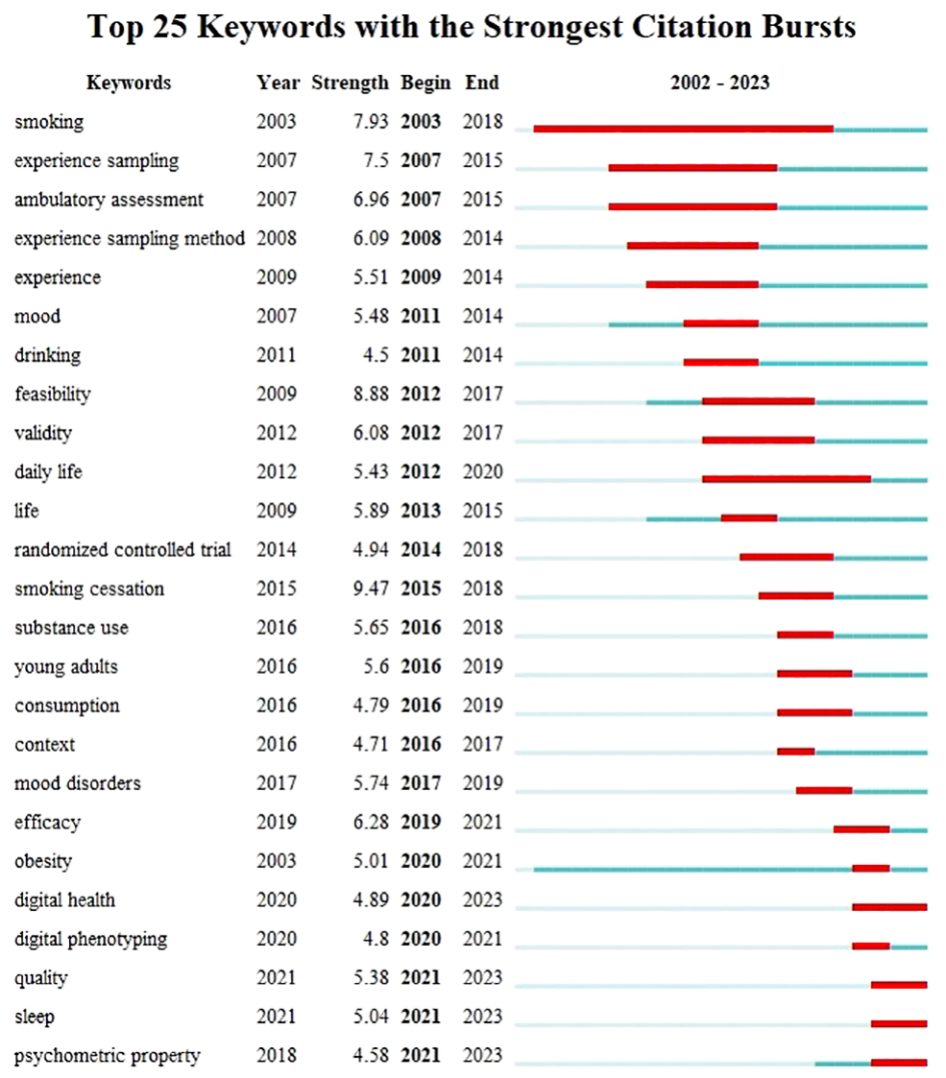
Top 25 Keywords with the most powerful Citation Bursts.

Keyword emergence refers to the rapid surge in the frequency of keywords at a certain point, reflecting the research frontier in the field. **Figure 9** displays the top 25 keywords with the strongest citation bursts spanning from 2003 to 2023. Since 2020, the terms “digital health”, “digital phenotyping”, “quality”, “sleep”, and “psychometric properties” have received the most attention, indicating possible future frontiers for research in mobile-based EMA and EMI.

Furthermore, an analysis of trending topics was conducted on 164 reviews within the selected literature, as depicted in **Supplementary Figure 2**. The findings reveal a concentrated focus on psychiatric disorders, notably bipolar disorder and schizophrenia, between 2017 and 2019. This focus shifted towards cognitive-behavioral therapies, anxiety, and depression from 2019 to 2021, among other areas pertinent to psychological disorders. Beyond 2021, the thematic emphasis of the reviews transitioned towards generalized disorders, symptoms, and the reliability of EMI approaches.

## 4 Discussion

### 4.1 Publication output and growth rate

Over the course of decades, EMA and EMI have undergone significant development, with their theories and technologies progressively maturing and being applied to an ever-expanding range of disciplines. In 1994, Stone and Shiffman introduced EMA as a method that could be performed using tiny mobile technologies (1). Since then, EMA has gradually evolved, and numerous books and articles have been written to describe the method. In 2008, a more comprehensive review of the rationale, design, methodology, and usage of EMA was provided by Shiffman (1). In 2010, Heron presented the first analysis of the benefits of mobile technology for EMI and reviewed the implementation of mobile-based EMIs across different disciplines. This provided valuable guidance for the future implementation of EMA and EMI using mobile technology and helped advance the use of EMA and EMI to a new stage (4). In the last decades, the utilization of EMA and EMI in areas including substance abuse, mental health, and physical activity began to grow significantly.

Furthermore, dynamically and individually tailored EMI emerged, also referred to as Just-in-Time Adaptive Intervention (JITAI) (4, 28). JITAI leverages mobile and sensing technologies to monitor participants’ ever-changing internal and contextual states in real-time (29). It offers flexible support delivery concerning time and location, providing timely and targeted interventions when they are most likely to be received and effective (29, 30). JITAI has been increasingly used to support health behavior interventions in various domains (28). The explosive growth in mobile-based EMA and EMI documents, fueled by advances like JITAI, demonstrates the enormous potential of this field. A concerted effort from diverse disciplines and professions will be critical to comprehensively grasp the potential of mobile-based EMA and EMI and drive future innovations in research and practice.

### 4.2 Leading journals

The journals JMIR mHealth and uHealth, JMIR, and JMIR formative research were the primary research sources on mobile-based EMA and EMI. With 209 documents published between 1992 and 2023, these journals offer a wealth of reliable literature. Scholars can use these journals to keep abreast of the latest research trends and findings. Moreover, the journals’ submission guidelines provide valuable references for researchers seeking to publish their work.

### 4.3 Leading countries and affiliations

The United States had the highest volume of mobile-based EMA and EMI publications, surpassing Germany, the second-highest-ranking country by over four times. The University of Pittsburgh and Penn State University, both leading institutions, are based in the United States, underlining its significant contributions to this field. Among the leading countries with the most scholarly publications in mobile-based EMA and EMI, developed countries played a primary role. It is notable that China, as the only developing country, has also contributed to this field’s development. Furthermore, the publication outputs of cooperative countries were lower compared to individual countries. Collaborative efforts among authors, countries, and institutions are crucial for advancing this research field. Therefore, it is recommended to continue promoting international collaborations among scholars and institutions to facilitate progress.

### 4.4 Research hotspots and trend topics

The first red cluster was centered around mental health, including depression, validity, stress, anxiety, mood, disorders, schizophrenia, and cognitive-behavioral therapy (CBT). Mental illness is a significant challenge in modern healthcare, with most individuals experiencing mental health problems at some point (31, 32). Mobile technologies’ utility in treating mental disorders is a significant component of the future of mental health management, and smartphones can enable patients to have a positive role in managing mental health syndromes and aiding in their recovery (33–35). By using mobile technology, EMA and EMI delivered through messaging, apps, and phone use can provide patients with much-needed psychological support in their daily lives. EMA is frequently used as a research tool to examine symptoms and potential mechanisms, and the reliability and efficacy have been established among this population (36–38). Additionally, There is an increasing interest in utilizing the EMA method for clinical purposes, including accurately assessing symptoms, identifying and monitoring signs of relapse, and tracking treatment effectiveness (39). CBT is a widely used form of psychotherapy effective in treating mental health conditions (40, 41). EMI using CTB is highly effective, particularly in the areas of depression (42, 43), anxiety (43), and schizophrenia (44). It also increases self-management abilities, satisfaction, positive emotions, and positive well-being and facilitates the development of learning skills (45). Our analysis of the theme map reveals that mental health is a rapidly developing and crucial area in mobile-based EMA and EMI research. It aligns with previous research consistently identifying it as the most prevalent theme (46), representing current and future research directions and hot topics.

The second blue cluster focused on behavior, physical-activity, time, exercise, consumption, smoking, obesity, and alcohol-use, which are associated with the role of mobile-based EMA and EMI in health behaviors. Physical activity, sedentary behavior, dietary behavior, smoking, and excessive alcohol consumption are critical health behaviors that can lead to the onset of diseases and premature death (47, 48). The primary characteristic of healthy behaviors is that they needs to be carried out consistently and regularly, ideally daily or several times per day, and sustained over the long term, even throughout an individual’s lifespan (49). However, maintaining healthy behaviors can be particularly challenging due to daily fluctuations in factors such as mood, social interactions, environmental obstacles, and location, making it challenging to maintain a consistent routine (49). EMA and EMI have emerged as effective tools for monitoring and managing health behaviors. EMA has been used in a broad spectrum of health behaviors (50–53), and study results provide a more accurate insight into how health behaviors change over time context (54). EMI has been implemented in the fields of smoking cessation (55), physical activity (56), alcohol cessation (57), and substance use (14) and has achieved some progress and effectiveness. However, some research results have yet to be significant, and it is hoped that future scholars will continue to explore and study (58, 59). The mobile-based EMA and EMI in health behaviors are promising and vigorously developing themes in the motor domain.

The third green cluster included feasibility, interventions, prevalence, reactivity and technology. Despite the numerous benefits of EMA and EMI, implementing these approaches can present several feasibility challenges. For example, frequent assessments may disrupt participants’ routines, potentially leading to lower adherence and increased dropout rates, particularly among patients with certain diseases (60, 61). In addition to these challenges, cost, participant attitudes, confidentiality concerns, and equipment loss or damage are other factors that must be addressed for successful implementation and patient acceptance (3). Another important consideration is the potential for reactivity or behavioral changes when study participants become aware that they are being assessed or intervened with. Reactivity could negatively impact the validity and accuracy of study results (4). Given these challenges and difficulties, it is important to identify key factors that can improve feasibility and compliance, and reduce reactivity. Future guidelines should systematically address these factors (61). Despite the challenges, advancements in mobile technology offer promise for enhancing the usability and acceptability of EMA and EMI approaches. It is a promising area for further development and exploration (3).

Additionally, the trend topics analysis specifically targeting reviews also uncovered some interesting findings. The initial reviews focused on the application of EMA and EMI within the field of psychiatry, primarily targeting bipolar disorder and schizophrenia. As the treatment of mental illnesses evolved, the application of EMA and EMI in cognitive-behavioral therapy also saw substantial development. Furthermore, EMA and EMI have been applied in the realm of psychological disorders, such as anxiety and depression. In recent years, EMA and EMI have been involved in symptom management across various diseases, thus shifting the review’s keywords towards more broadly defined terms such as “disorders” and “symptoms”. With the widespread application of EMA and EMI, their reliability has also got great attention, giving rise to numerous papers and reviews in this field. Unlike previous reviews that focused on specific areas related to EMA and EMI, this article, as a review utilizing bibliometrics, explores more broadly the development trajectory, application domains, and current state of EMA and EMI, serving as a summary and supplement to existing literature.

Currently, mobile-based EMA and EMI have shown significant progress in multiple fields. They also expand gradually to other health domains (23). Additionally, there has been a consistent emphasis on tailoring EMA and EMI as a trend. Tailoring EMI based on EMA is a new research area that can increase user engagement and intervention effectiveness. However, its efficacy is still to be verified, and robust clinical trials will be needed to test tailoring EMI based on EMA in different populations and settings (46). The era of implementing interventions using Just-In-Time Adaptive Interventions (JITAI) is approaching. A key research direction for the future is to develop sophisticated and intricate health behavior theories to guide the design and delivery of such interventions while implementing them (28). Another crucial trend is the emergence of digital phenotyping, which, aided by machine learning, generates ecological, continuous, and personalized digital phenotypes to enhance diagnostic and evaluative accuracy based on EMA data collected from smartphones, wearables, and human-computer interactions (61–64). The advent of digital tools may pose unprecedented challenges to clinical and subjective assessments by clinicians and questionnaires. It underscores the potential for digital tools to bring about revolutionary breakthroughs and developments in mental health and healthcare. Achieving this will require collaborative efforts from interdisciplinary teams in the future (65).

### 4.5 Limitations

Although the research offers valuable perspectives on EMA and EMI in mobile technology literature from 1992 to 2023, several limitations should be considered. Firstly, our study only searched the WOS database, which may not provide a complete picture of all relevant publications. Including additional databases like Scopus and PubMed could yield a more comprehensive view of the literature. Furthermore, despite our best efforts to ensure the precision and comprehensiveness of our search, there may still be omissions in our selection of keywords. Consequently, the results are inevitably influenced by the selected keywords. Additionally, the data on citations may be subject to temporal limitations, with papers published earlier often having a higher citation frequency than those published more recently. This could result in some influential papers not receiving the attention they deserve simply because they were published later. Moreover, the studies included in this article are published in English, which might introduce a language bias into the bibliometric analysis. This could lead to the omission of significant research published in other languages, thereby making the analysis less comprehensive. Finally, our analysis only covers publications until the search date, which may limit the validity of our findings. Future updates may be necessary to capture new developments in the field. Finally, to achieve a deeper overview of the current state and emerging research frontiers, it may be beneficial for future studies to engage and collaborate with experts in the field.

## 5 Conclusion

Our study employed bibliometric methods to provide a bird’s eye view of mobile-based EMA and EMI research, offering valuable insights for scholars to understand these technologies evolution, development, and future trends. Our analysis of the most productive and influential authors, countries, and core journals has guided researchers in selecting journals to submit to and potential research partners to collaborate with. Furthermore, in the analysis of the hot topics, we found that mobile-based EMA and EMI show great potential in the fields of health behaviors and mental health. However, it is necessary to rigorously and carefully consider the feasibility and reactivity issues during the implementation process. In the future, mobile-based EMA and EMI could be expanded to other health domains, and research on EMA-tailored EMI, JITAI and the incorporation of wearable devices, machine learning, and digital phenotyping are emerging trends. This study offers a valuable reference for scholars to understand the evolution, development, and future trends of EMA and EMI in mobile technology and inform their future research choices.

## Data availability statement

The original contributions presented in the study are included in the article/**Supplementary Material**, further inquiries can be directed to the corresponding author/s.

## Author contributions

HY: Conceptualization, Data curation, Methodology, Writing – original draft, Writing – review & editing. HZ: Conceptualization, Methodology, Writing – original draft. JG: Data curation, Writing – original draft. HQ: Data curation, Writing – original draft. WD: Data curation, Methodology, Writing – review & editing. NG: Data curation, Methodology, Supervision, Writing – review & editing. JF: Conceptualization, Data curation, Methodology, Supervision, Writing – review & editing. YY: Conceptualization, Data curation, Methodology, Supervision, Writing – review & editing.
