# Supplementary figures and images for: Mobile-based ecological momentary assessment and intervention: bibliometric analysis
Source: Front Psychiatry. 2024 Feb 26;15:1300739. doi: 10.3389/fpsyt.2024.1300739 (PMC10925651; doi:10.3389/fpsyt.2024.1300739)

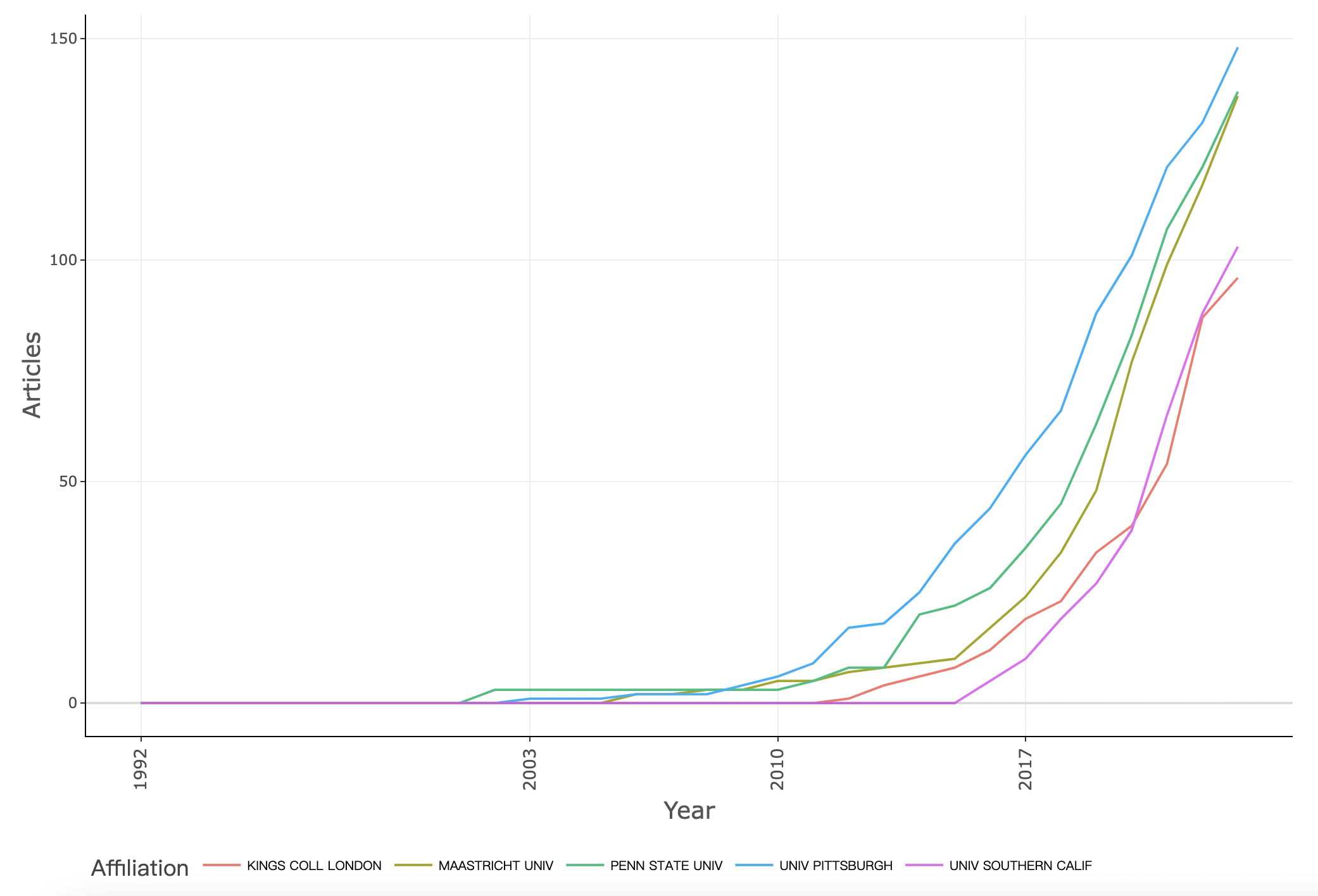

Supplement: Supplementary file 1 [file Image_1.jpeg]

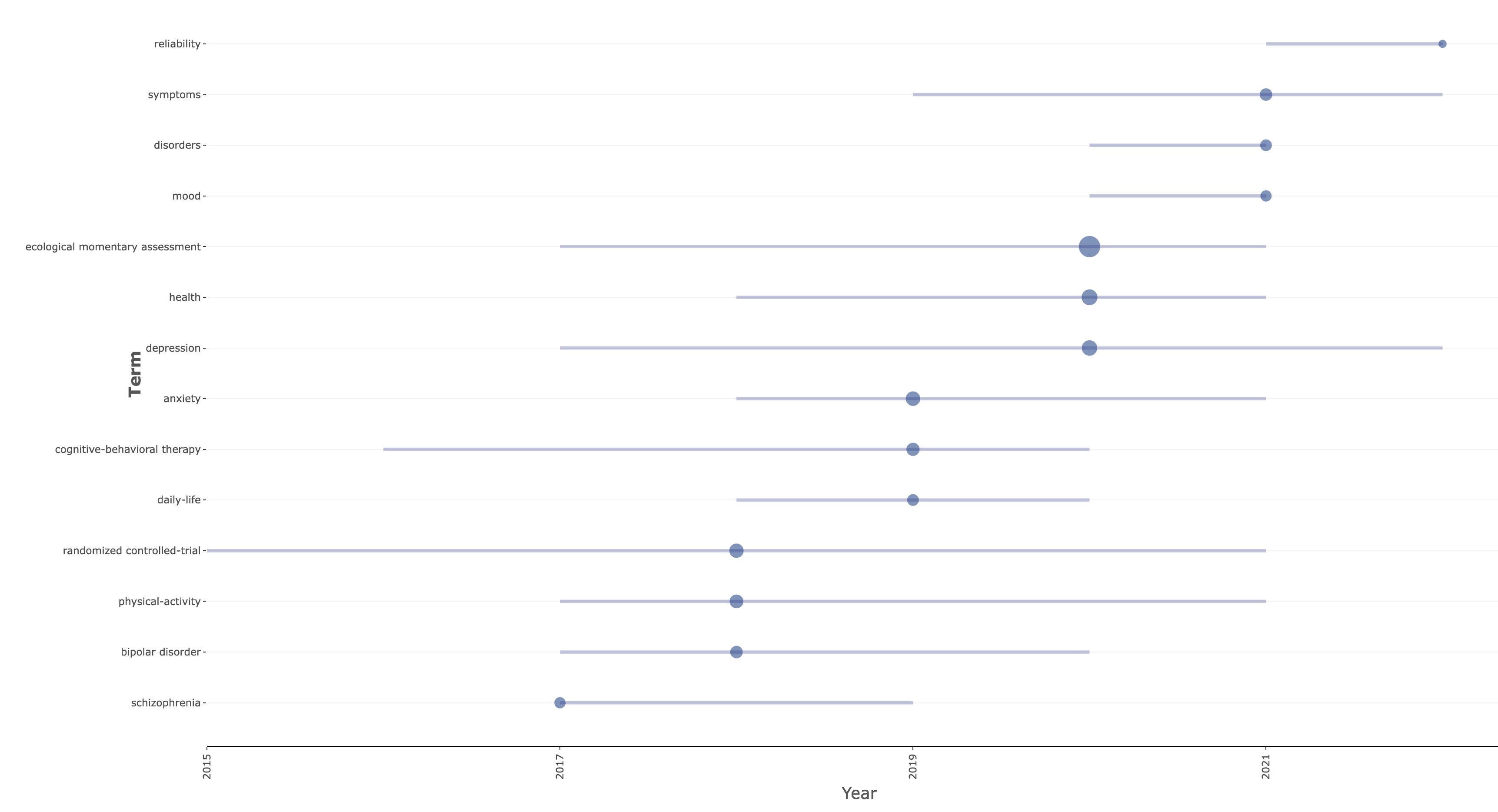

Supplement: Supplementary file 2 [file Image_2.jpeg]
